# Satellite tracking of rehabilitated sea turtles suggests a high rate of short-term survival following release

**DOI:** 10.1371/journal.pone.0246241

**Published:** 2021-02-16

**Authors:** David P. Robinson, Kevin Hyland, Gerhard Beukes, Abdulkareem Vettan, Aneeshkumar Mabadikate, Rima W. Jabado, Christoph A. Rohner, Simon J. Pierce, Warren Baverstock

**Affiliations:** 1 Dubai Turtle Rehabilitation Project, Dubai, UAE; 2 Sundive Research, Byron Bay, NSW, Australia; 3 Marine Megafauna Foundation, Truckee, CA, United States of America; 4 Wildlife Protection Office, Dubai, UAE; 5 Jumeirah Group, Dubai, UAE; 6 Elasmo Project, Dubai, United Arab Emirates; 7 Amaala, Riyadh, Kingdom of Saudi Arabia; Deakin University, AUSTRALIA

## Abstract

The rehabilitation of wildlife can contribute directly to the conservation of threatened species by helping to maintain wild populations. This study focused on determining the post-rehabilitation survival and spatial ecology of sea turtles and on comparing the movements of individuals with flipper amputations (amputees) to non-amputee animals. Our aims were to assess whether rehabilitated sea turtles survive after release, to compare and contrast the movement characteristics of the different species of sea turtles we tracked, and to examine whether amputees and non-amputees within species behaved similarly post-release. Twenty-six rehabilitated sea turtles from four species, including hawksbill *Eretmochelys imbricata* (n = 12), loggerhead *Caretta caretta* (n = 11), green *Chelonia mydas* (n = 2), and olive ridley *Lepidochelys olivacea* (n = 1) sea turtles from the United Arab Emirates were fitted with satellite tags before release. Rehabilitation times ranged from 89 to 817 days (mean 353 ± 237 days). Post-release movements and survival were monitored for 8 to 387 days (mean 155 ± 95 days) through satellite tracking. Tag data suggested that three tracked sea turtles died within four days of release, one after 27 days, and one after 192 days from what are thought to be anthropogenic factors unrelated to their pre-rehabilitation ailments. We then compared habitat use and movement characteristics among the different sea turtle species. Although half of all turtles crossed one or more international boundaries, dispersal varied among species. Loggerhead turtles had a high dispersal, with 80% crossing an international boundary, while hawksbill turtles displayed higher post-release residency, with 66% remaining within UAE territorial waters. Amputee turtles moved similarly to non-amputee animals of the same species. Loggerhead turtles travelled faster (mean ± sd = 15.3 ± 8 km/day) than hawksbill turtles (9 ± 7 km/day). Both amputee and non-amputee sea turtles within a species moved similarly. Our tracking results highlight that rehabilitated sea turtles, including amputees, can successfully survive in the wild following release for up to our ~one-year monitoring time therefore supporting the suitability for release of sea turtles that have recovered from major injuries such as amputations. However, more broadly, the high mortality from anthropogenic factors in the Arabian Gulf region is clearly a serious issue and conservation challenge.

## Introduction

Around the world, the human population is rapidly increasing and technological advancements have reduced biological constraints on individuals, simultaneously increasing anthropogenic factors that negatively affect wildlife [1]. To reduce these growing impacts on species, a number of conservation tools have been used including the rehabilitation of wildlife [2–5]. Wildlife rehabilitation has been defined as providing temporary care, as necessary, to wild animals with the goal of eventual release back into wild [2]. Following the success and efficacy of programs aimed at enhancing populations of birds and mammals [6], this approach has become key in the conservation of threatened species, particularly terrestrial animals, and the preservation of biodiversity in both developed and developing countries. While rehabilitation needs to consider cultural and economic factors at local and regional levels, it also plays a vital role in increasing awareness of animal welfare issues at various scales [7]. Indeed, outreach efforts by rehabilitation centers can contribute to public awareness and education on threats, and their mitigation, and promote lasting changes for the benefit of the species they work with [5]. However, while Wimberger *et al*. [3] note the importance of post-release monitoring to assess the successes and failures of rehabilitation methods and highlight the need for ongoing research into protocols and characteristics to aid in the ability of rehabilitators to predict the survival of animals after they have been released.

Sea turtles have a low natural survival probability from egg to maturity. No parental care is afforded to hatchlings and, they are continually threatened throughout their life cycle by anthropogenic factors such as pollution and fisheries [8]. This makes the rehabilitation of individuals that have survived the high initial mortality rates helpful to the contribution of population stability or growth [8], particularly if they can then enter or re-enter the breeding population. While many injuries from anthropogenic origins can threaten individual sea turtles, rehabilitation can often save injured animals that would otherwise die; for example, entanglement in discarded monofilament line and fishing nets can cause serious wounds and amputations that can lead to death without treatment [4]. Furthermore, individual rescue and rehabilitation can be particularly important within small, threatened populations, such as hawksbill turtles *Eretmochelys imbricata* (Linnaeus 1766) in the Arabian Gulf region, which have low contemporary breeding numbers and low genetic variability [9]. In fact, the north-west Indian Ocean has been highlighted as a ‘High Risk, High Threat’ Regional Management Unit for hawksbill and olive ridley *Lepidochelys olivacea (*Eschscholtz 1829) turtles, as well as an area with critical data shortages [10], making it an ideal location to develop and implement rehabilitation programs.

Five of the seven sea turtle species have been documented in the Arabian Gulf (hereafter referred to as the ‘Gulf’): the hawksbill turtle, olive ridley turtle, green turtle *Chelonia mydas* (Linnaeus 1758), loggerhead turtle *Caretta caretta* (Linnaeus 1758), and leatherback turtle *Dermochelys coriacea* (Blainville 1816) [5, 6]. Hawksbill and green turtles nest in the Gulf, while loggerhead, olive ridley, and leatherback turtles are infrequent visitors that breed in the wider Indian Ocean [11]. The Gulf region has undergone rapid economic growth, involving substantial construction along coastal and offshore regions, underpinned by its massive oil and gas industry and by wealth derived from financial centers [12]. Ongoing threats to sea turtles in the Gulf include fisheries, urban and industrial development, direct hunting, oil exploration, shipping, and climate change [13]. Because of their vulnerability to natural physiological stressors in this environment, which is subject to extreme temperature changes on a seasonal basis, as well as these additive anthropogenic threats (boat strike, habitat loss), coupled with the financial resources available, sea turtles in the Gulf have proven to be excellent candidates for incorporating rescue, rehabilitation, and release as a conservation strategy [5].

Limited prior data are available on the movements and survival rate of rehabilitated sea turtles [5], and results have been mixed. Cardona *et al*. 2012 [14] satellite tracked 18 loggerhead turtles off the coast of Spain, six of which had been through extended periods of rehabilitation. Compared to 12 wild “control” sea turtles, four of the rehabilitated sea turtles showed anomalies in one or more of the tested behavioural parameters. Polovina *et al*. [15] found that the dispersal behaviour of captive-raised loggerheads released off Japan did not differ from wild individuals and Flegra *et al*. [16] found that a rehabilitated sea turtle tagged and released in the Mediterranean frequented known sea turtle foraging or breeding locations. Mestre *et al*. [8] tracked three rehabilitated sea turtles, one of which had the front flipper amputated, and found that, prior to release, the amputation had little effect on this individual’s swimming ability. After release, the animal was tracked for approximately 24 months and behaved similarly to the non-amputee animals included in the study.

Amputee turtles, or turtles requiring amputations after becoming entangled in discarded waste, have increasingly been received by the Dubai Turtle Rehabilitation Project (DTRP). The DTRP dataset provides an opportunity to expand knowledge of post-release survival for both amputees and non-amputees and aid rehabilitation projects with future release decisions. Our aims were: 1) to assess whether rehabilitated sea turtles survive after release, 2) to compare and contrast the movement characteristics of the different species of sea turtles we tracked, and 3) to examine whether amputees and non-amputees within species behaved similarly post-release. Here, we report on satellite tracking results from four sea turtle species, including six individuals with amputated flippers, focusing on their movements, thermal preferences, and spatial ecology, following their release after rehabilitation.

## Materials and methods

The Dubai Turtle Rehabilitation Project (DTRP) has been operating in its current form since 2004. To date, the DTRP has rehabilitated over 1,800 sick and injured sea turtles from around the Gulf region that have been released back into the wild [5]. This has involved a substantial investment in staff, facilities, and medical resources. In order to justify the financial expense of such programs, and particularly to improve long-term outcomes for the rescued animals, it is important to assess the success of rehabilitation programs [6]. Sea turtles are amenable to remote tracking through the attachment of satellite tags to their carapaces, and this technique provides the opportunity to assess post-release behavior and survival in the DTRP’s rehabilitated and released sea turtles [5]. Satellite tracking has provided insights into the behaviors and spatial ecology of many species, including sea turtles [17], and can also help to identify critical habitats for management purposes [18–21].

### Study area

The Arabian Gulf covers an area of 230,000 km^2^ and lies in the subtropical northwest of the Indian Ocean between latitudes of 24°N and 30°N and longitudes of 48°E and 57°E [12]. It is a shallow, almost enclosed sea with an average depth of 30 m. The Gulf is the warmest sea in the world [12], and environmental conditions within this body of water are among the most extreme on the planet [22]. Air temperatures in the region can drop to 0°C in winter and reach in excess of 50°C in summer, strongly influenced by the prevailing winds [12] while sea surface temperature fluctuates from 10°C in winter to up to 39°C in summer [23]. The Gulf has limited water exchange with the Gulf of Oman through the Strait of Hormuz and Musandam area of Oman to the northeast. The only freshwater input to the Gulf enters via the Shatt al Arab estuary by the Euphrates and Tigris rivers [12]. Despite these conditions, the Gulf is still a highly productive sea that harbors extensive sea grass beds, mangroves, salt marshes, and coral reefs [12].

### Study animals

Permissions for sea turtle rehabilitation work were given by the Dubai Wildlife Protection Office with whom this work was conducted. Twenty-six sea turtles were brought to the Dubai Turtle Rehabilitation Project between March 2012 and January 2018 suffering from various ailments including cold stunning (n = 3), debilitating physical injuries such as boat impact trauma (n = 4), general debilitation (n = 7), and infection, such as those described in Caliendo et al., [24] (n = 6). An additional six sea turtles were admitted with one front flipper already missing, due to injury, or had a flipper amputated by a veterinarian due to damage caused by entanglement in discarded waste. Rehabilitated sea turtles were from four different species, including 12 hawksbill, 11 loggerhead, two green, and one olive ridley.

Different species of sea turtles enter different life stages at different sizes [25]. Sea turtles within this study were classified as either juvenile, sub-adult, or adult based on species-specific curved carapace lengths (CCL). In Qatar, hawksbills were immature at 21 cm CCL and one adult was mature at 72 cm CCL. Hawksbills from the UAE were mature at a similar size of 70.3cm CCL [13]. No regional data exist on the size of subadults. We therefore classified hawksbill turtles as juvenile (<55 cm), sub-adult (>55–70 cm) or adult (>70cm). One sub-adult loggerhead was measured at 68.5 cm CCL [25], and loggerheads from Oman were mature at 94 cm CCL [26]. We therefore classified loggerhead turtles as juvenile (<50 cm), sub-adult (50–70 cm) or adult (>70 cm). Olive ridley turtles regionally mature above 71 cm CCL [27] and the single individual in this study had a CCL of below 60 cm and so was classed as a sub-adult. The included green turtle was classified as adult (>90 cm) based on size data by Gasperetti *et al*. [28]. All adult sea turtles were female based on their short tails, as described in Robinson et al., [5]. Juvenile and sub-adult individuals were not sexed.

### Tag deployment

Sea turtles were released between May 2012 and May 2018 and were tracked using SPOT-5 back-mount tags from Wildlife Computers (Seattle, USA). These tags uplink to the Argos satellite system (http://www.argos-system.org) whenever they break the water surface and a satellite is overhead. Tags were attached and programmed using the protocols described in Robinson et al., [5]. In brief, slow hardening and low heat-generating epoxy was used for attachment and covered with coatings of antifoul paint in accordance with the tag manufacturer’s instructions. To save battery power, and increase track duration, tags were restricted to 250 transmissions per day and programmed to transmit during the daylight hours (06:00–18:00) only. Apart from messages to allow the Argos system to determine location, the SPOT-5 tags also transmitted temperature information. Seven 3°C bins ranging from 18°C to 39°C were used to record daily percentages of time-at-temperature for each sea turtle. Haul out mode was activated to record if the tag spent any time outside the water.

Three of the rehabilitated sea turtles were killed by boat strike within a week of release which was visually confirmed through the recovery of the animals. These tracks were excluded from the tracking analyses (Table 1). Two other sea turtles suffered mortality after more than a week at liberty, these tracks were included in the analyses as they contributed to the data (Table 1).

**Table 1:** A summary of the rehabilitated sea turtles that suffered post-release mortality.

Sea turtle Name	Species	Life Stage	Tracking Analysis	Ailment on Admission	Rehabilitation Duration (days)	Release Weight (kg)	Curved Carapace Length (cm)	Tracking Duration (days)
**Spots**	*C*. *mydas*	Sub-adult	Excluded	Amputee	730	41	79	<4
**Lucky**	*E*. *imbricata*	Adult	Excluded	Amputee	756	30	84	<4
**Olive**	*L*. *olivacea*	Sub-adult	Excluded	Debilitation	390	19	57	<2
**Aqua**	*E*. *imbricata*	Sub-adult	Included	Amputee	700	25.5	63	27
**Kruneloni**	*C*. *caretta*	Sub-adult	Included	Infection	159	34	70	192

### Satellite-data filtering and analysis

Argos transmitters use standard Doppler-based geolocation technology. Locations were assigned an estimate of accuracy by the Argos system: class 0 = >1,500 m, Class 1 = >1,000 m, class 2 = >500 m, and class 3 = >150 m, with class A and B given no estimate of accuracy. To collect and organize data, we used the Satellite Tracking and Analysis Tool (STAT) [29] prior to 2017, and the Wildlife Computers data portal (www.wildlifecomputers.com) after that.

When tags stopped transmitting, diagnostic data such as previous movements and strength and frequency of signals were monitored as described in Hays et al., [30]. In addition, percentage of dry time and temperature histogram data were investigated to establish if the cause of transmission failure was linked to suspected mortality or tag failure. For example, if a tag had reported normally and then stopped all transmissions, we assumed that the sea turtle was still alive, but the tag’s antenna was damaged or overgrown, or the tag itself was damaged or fell off. In this case it is also possible that the sea turtle died and floated upside down, or that it lost its tag due to a lethal injury. Two of the dead sea turtles were physically recovered with their tag missing due to the impact of the boat strike. Apart from such observed mortality, we assumed sea turtle mortality when the tag started to continuously transmit high-quality location data, indicating that the tag, and the dead sea turtle, were floating at the surface. Detached tags would sink and not transmit. We confirmed this assumption when we recovered one tagged sea turtle that was killed by boat strike leaving the tag intact and applied the same method to the two dead sea turtles that were not recovered but exhibited the same pattern of transmissions. None of the tags on live sea turtles indicated a similar pattern of continuous transmissions.

The Douglas filter was applied to all data in Movebank (www.movebank.org) [31] to improve location accuracy and remove outlying data, following the protocol in Robinson et al., [5]. Locations with duplicate timestamps were automatically removed. Locations assigned Argos classes 1, 2 or 3 were retained in the analysis. Argos locations with a B grade and above were also included but could be excluded by the filter if outside the specified parameters. The Douglas filter used a maximum redundancy distance of 10 km, a maximum realistic rate of movement of 5 km / hour, and a turning angle filter of 25°, as in Robinson et al., [5]. The Douglas filter removed a mean of 6 ± 5% of outlying data (n = 28; median = 5%; range 1–22%). Horizontal track distance was calculated as the sum of the shortest straight-line distances between consecutive locations, and thus did not include any vertical movement component.

To examine the overall displacement of a sea turtle we calculated the total distance travelled, and to assess travel speed we calculated the daily distance covered in km per day. For example, a sea turtle that displayed high residency far away from the release location had a high dispersal but a low travel speed.

We tested normality with a Shapiro test and compared means between groups with a t-test for normally distributed data and with a Mann-Whitney test for non-normally distributed data. When one group only had one data point (e.g. amputee loggerhead turtles), we assumed equal variance for the two groups when testing significance.

### Home range analysis

Data were exported from Movebank and imported into ArcGIS 10.2.1 for spatial analyses using the methodology outlined in MacLeod [32]. In brief, the “Spatial Analyst” and “Reclassify” Tools were used to calculate home range or 95% Percentage Volume Contour (PVC), and core habitat area or 50% PVC. To calculate the overlap of individual home ranges within each species, we used the “Raster Calculator” and “Map Algebra” tools. We extracted bathymetric depths at all locations using depth contours in ARCGIS 10.2.1 to investigate the preferred depth range for each turtle species.

### Temperature analysis

We calculated the percentage of time spent in each 3°C temperature bin on a monthly basis and compared the distribution for amputee and non-amputee sea turtles for months when temperature data were transmitted and comparable, which excluded September, October, and November.

## Results

### Tracking

#### Hawksbill turtles

The tracking duration for 11 hawksbill turtles ranged from 27 to 263 days (mean = 131 ± 67 days; median = 123) and distances covered ranged from 62 to 3,033 km (mean = 1,101 ± 960 km; median = 632 km). Home ranges (95% PCV) for hawksbill turtles ranged between 25 and 7,086 km^2^ (mean = 1,447 ± 2,415 km^2^; median = 332 km^2^; Table 2).

**Table 2:** A summary of the rehabilitation information and resulting tracking data for all hawksbill turtles in this study.

Sea Turtle Name	Life Stage	Ailment on receipt	Rehab duration (days)	Curved Carapace Length (cm)	Release Weight (kg)	Tracking Duration (days)	Distance covered (km)	Distance Travelled (km/day)	MBG AREA (km^2^)	Mean Depth (m) (min-max)	Home Range (PVC 95%, km^2^)	Core habitat (PVC 50%, km^2^)	Maritime Boundaries
**Noor**	Juvenile	Debilitation	300	48	12.1	156	622	4	5628	9 (3–19)	805	150	UAE
**Torpedo**	Sub-adult	Cold Stunned	89	49	13.5	123	1413	11	477	12 (9–17)	153	25	UAE
**Mashuwa**	Juvenile	Debilitation	340	50	13.7	77	433	6	4907	13 (5–27)	240	39	UAE
**Shadeed**	Sub-adult	Debilitation	250	53	15.62	118	507	4	561	10 (7–11)	59	15	UAE
**Crush**	Juvenile	Cold-stunned	100	54	16.95	68	480	7	4682	10 (39–99)	221	56	UAE / OMAN
**Jameel**	Sub-adult	Debilitation	314	55	15.8	78	62	1	50	6 (4–8)	25	6	UAE
**Juzasu**	Sub-adult	Cold-stunned	97	57	17.2	171	2517	15	13825	34 (5–87)	5354	413	UAE / IRAN
**Seabiscuit**	Sub-adult	Injury	423	64	23.7	166	632	4	854	12 (4–20)	477	102	UAE
**Leonardo**	Sub-adult	Injury	406	65	27	193	1774	9	14992	16 (9–23)	332	83	UAE / QATAR
**Oogie**	Sub-adult	Amputee	817	71	33.5	263	3033	12	29097	54 (1–101)	7086	704	IRAN / UAE
**Aqua**	Sub-adult	Amputee	700	63	25.5	27	641	24	71752	35 (9–96)	1162	271	UAE / OMAN
**Mean**			**349 ± 236**	**57 ± 8**	**20 ± 7**	**131 ± 67**	**1101 ± 960**	**9 ± 7**	**11773 ± 21749**	**19 ± 15**	**1447 ± 2415**	**169 ± 217**	**NA**

MBG = Minimum Bounding Geometry, PVC = Percentage Volume Contour.

Hawksbill turtles swam 8.8 km day^-1^ on average, with amputee sea turtles travelling faster (17.6 ± 8.6 km day^-1^, n = 2) than non-amputees (6.8 ± 4.3 km day^-1^, n = 9; t = -2.8, df = 9, p = 0.02). The overall track distance for amputee hawksbill turtles ranged from 641 to 3,033 km (mean = 1,837 ± 1,691 km; n = 2), and for non-amputee hawksbill turtles ranged from 62 to 2,517 km (mean = 938 ± 793 km; n = 9). Amputee hawksbill turtles had a similar track duration (mean ± SD = 145 ± 166.9 days, n = 2) to non-amputees (128 ± 46.3 days, n = 9; t = -0.31, df = 9, p = 0.76).

Home range areas varied widely among individuals. Amputees had extent home ranges of 1,162 km^2^ and 7,086 km^2^, while non-amputees had extent home ranges from 25 km^2^ to 5,354 km^2^ (mean = 852 ± 1,705 km^2^; n = 9) (Table 2). Notably, amputee hawksbill turtles occupied areas with a deeper mean depth (44.5 m) than non-amputees (19 m).

#### Loggerhead turtles

Tracking durations for the 11 loggerhead turtles ranged from 22 to 387 days (mean = 161 ± 103 days; median = 15) and distances covered ranged from 150 km to 5,060 km (mean = 2,339 ± 1,411 km; median = 2,178 km). The amputee loggerhead turtle had a similar track duration (80 days) to non-amputees (169.4 ± 104.4 days, n = 10; t = 0.82, df = 9, p = 0.44). Home ranges for loggerhead turtles ranged between 254 and 7,941 km^2^ (mean = 2,537 ± 2,545 km^2^; median = 1,242 km^2^; Table 3).

**Table 3:** A summary of the rehabilitation information and resulting tracking data for tracked loggerhead turtles.

Sea turtle Name	Sex / Life Stage	Ailment on receipt	Rehab Duration (days)	Curved Carapace Length (cm)	Release Weight (kg)	Tracking Duration (days)	Distance covered (km)	Distance Travelled (km/day)	MBG AREA (km^2^)	Mean Depth (m) (min-max)	Home Range (PVC 95%, km^2^)	Core habitat (PVC 50%, km^2^)	Maritime Boundaries
**Cousteau**	Unknown/ Juvenile	Debilitation	119	24	1.5	22	150	7	11408	39 (14–69)	901	163	UAE
**Storm**	Female/ Adult	Infection	122	95	95	170	4643	27	48567	62 (4–81)	1210	683	UAE / QATAR / IRAN
**Kruneloni**	Unknown/ Sub-adult	Infection	159	70	34	192	2919	15	96605	31 (9–79)	5157	921	UAE / IRAN / QATAR / KUWAIT / IRAQ
**Joey**	Female/ Adult	Infection	412	74	63.5	92	907	10	7185	46 (44–204)	2066	45	UAE / OMAN
**Mojah**	Female/ Adult	Infection	200	86	64.8	214	2712	13	92800	20 (4–55)	1030	369	UAE
**Schwab**	Female/ Adult	Infection	304	90	70.28	155	1769	11	6105	36 (9–89)	1242	65	UAE / OMAN
**Salem**	Unknown/ Sub-adult	Debilitation	134	69	33.4	78	1960	25	34505	56 (2–89)	1745	538	UAE / OMAN / IRAN
**Turtala**	Female/ Adult	Injury	400	84	54	266	4099	15	17962	7 (1–20)	587	306	UAE
**Rosie**	Female/ Adult	Injury	133	80	50	118	1170	10	3413	29 (9–42)	254	63	UAE
**Sam**	Female/ Adult	Infection	120	85	56	387	2809	7	43485	15 (2–55)	7941	741	UAE / QATAR
**Beau**	Female/ Adult	Amputee	703	97	95	80	2178	27	71752	55 (9–89)	5774	1021	UAE / IRAN / QATAR / SAUDI ARABIA
**Mean**			**255 ± 186**	**78 ± 20**	**56 ± 27**	**161 ± 103**	**2301 ± 1334**	**15 ± 8**	**39435 ± 34612**	**36 ±18**	**2537 ± 2545**	**447 ± 356**	**NA**

MBG = Minimum Bounding Geometry, PVC = Percentage Volume Contour.

One amputee loggerhead turtle (‘Beau’) and 10 non-amputee loggerhead turtles were included in the comparison tracking and spatial ecology analysis. The amputee loggerhead turtle had a similar track distance (2,178 km) to the mean of the non-amputees (mean = 2,355 ± 1,486 km;). Amputee (27.2 km day^-1^) and non-amputee loggerhead turtles (14.1 ± 7.02 km day^-1^) travelled at a similar speed (W = 9, p = 0.36). The home range for the amputee loggerhead turtle was 5,774 km^2^. Home ranges for non-amputee loggerheads ranged from 254 to 7,941 km^2^ (mean = 2,213 ± 2,433 km^2^; median = 1,226). The mean depth of the home range for non-amputee loggerheads ranged from 7 to 62 m (mean = 34 ± 18 m). The distance covered, home range, and mean depth (55 m) for the amputee loggerhead all fell within the range of the non-amputee loggerheads.

One amputee green turtle (Al Ouda) was included in the tracking and spatial ecology analysis. The distance covered for the amputee green was 2,320 km over 141 tracking days. The home range for the amputee green turtle was 535 km^2^ and the mean depth was 11 m (Table 4).

**Table 4:** A summary of the rehabilitation information and resulting tracking data for a tracked amputee green turtle.

Sea turtle Name	Sex / Life Stage	Ailment on receipt	Rehab Duration (days)	Curved Carapace Length (cm)	Release Weight (kg)	Tracking Duration (days)	Distance covered (km)	Distance Travelled km/day	Mean Depth (m) (min-max)	MBG AREA (km^2^)	Home Range (PVC 95%, km^2^)	Core habitat (PVC 50%, km^2^)	Maritime Boundaries
**Al Ouda**	Female / Adult	Amputee	653	98	92	141	2320	16.45	31 (1–11)	11382	535	25	UAE / QATAR

MBG = Minimum Bounding Geometry, PVC = Percentage Volume Contour.

### Horizontal movements

#### Hawksbill turtles

**Fig 1:**
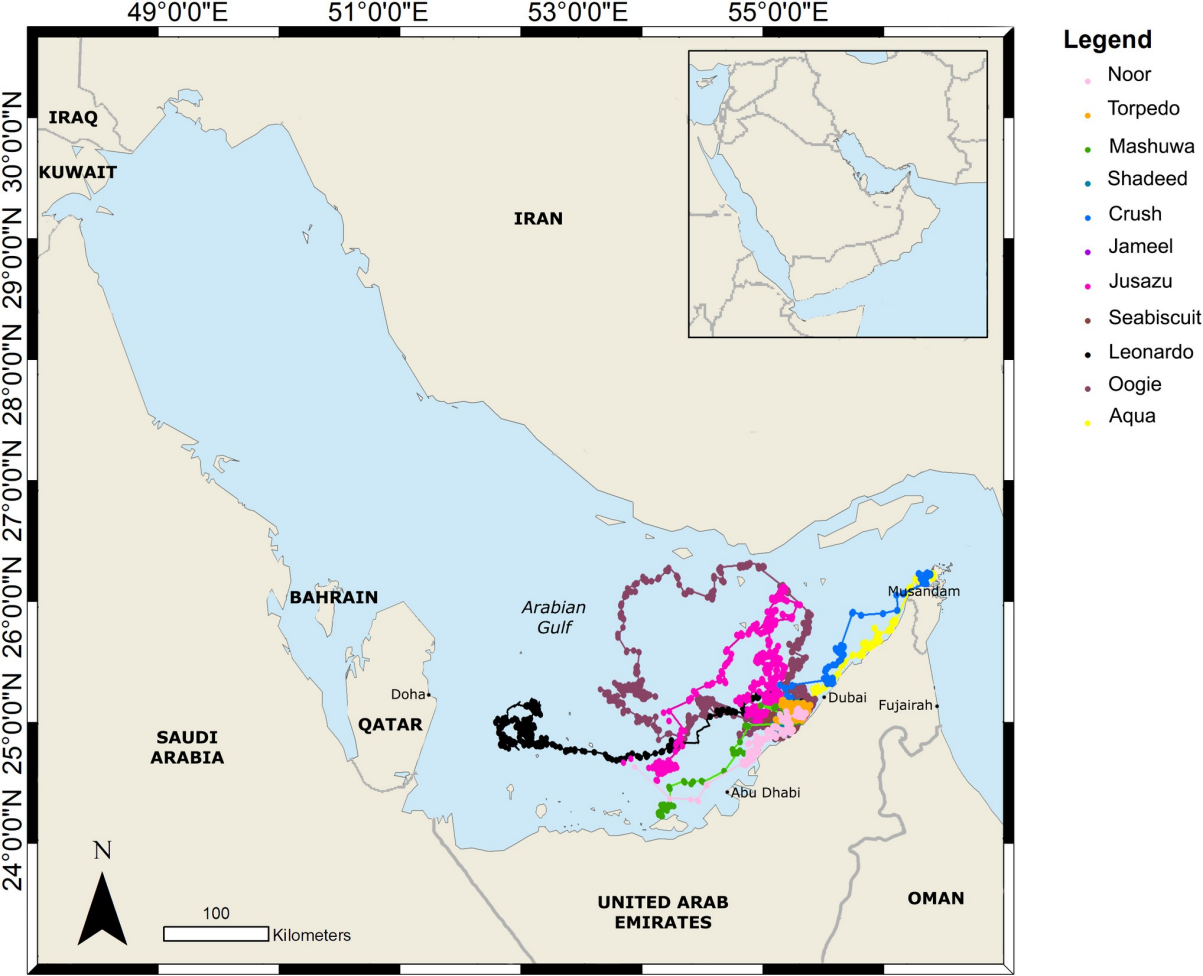
Horizontal movements of eleven rehabilitated hawksbill turtles satellite tagged and released in the UAE during this study.

Rehabilitated hawksbill turtles displayed large individual variation in their horizontal movements after release. Six hawksbill turtles remained in UAE waters throughout their tracked movements (Fig 1). They each formed their core habitat areas between Dubai and Abu Dhabi. Other hawksbill turtles moved further afield, including amputee ‘Oogie’ that travelled across the Gulf and into Iranian waters before returning back to form its core habitat area close to the release point in Dubai (Fig 1). Amputee ‘Crush’ was the only hawksbill to travel north into Omani waters and towards the Strait of Hormuz. Its core habitat area was formed off the town of Khasab.

#### Loggerhead turtles

**Fig 2:**
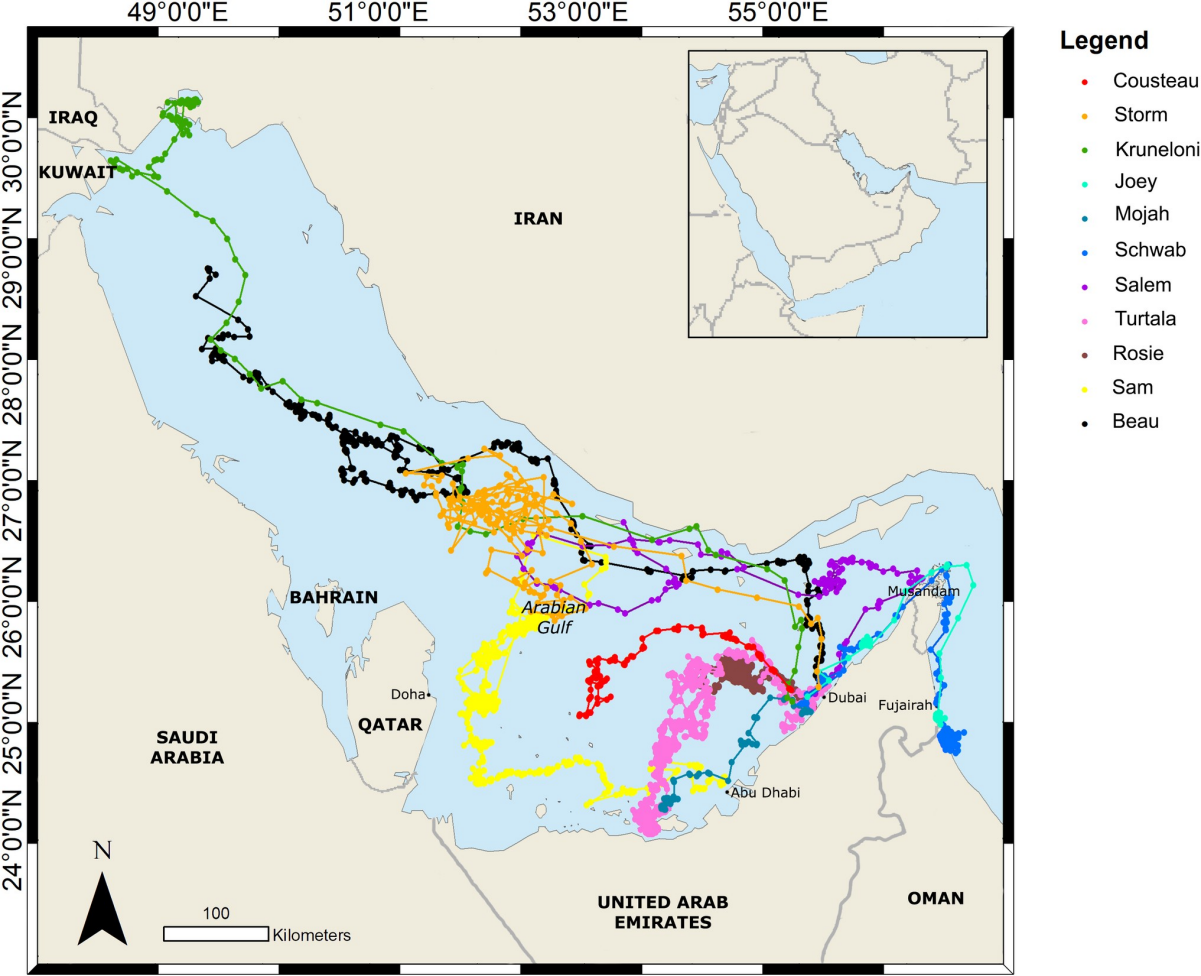
Horizontal movements of eleven rehabilitated loggerhead turtles satellite tagged and released in the UAE during this study.

Loggerhead turtles dispersed widely into the Gulf, with two sea turtles ‘Joey’ and ‘Schwab’ even exiting the Gulf through the Strait of Hormuz and entering the Gulf of Oman (Fig 2). On 28 September 2012, Kruneloni’s tag suddenly started transmitting many high-class locations and then stopped transmitting for a period of weeks. The tag then started transmitting again on 20 November 2012 with many high-class locations commonly associated with a tag being permanently exposed to air, suggesting possible mortality.

#### Green turtle

**Fig 3:**
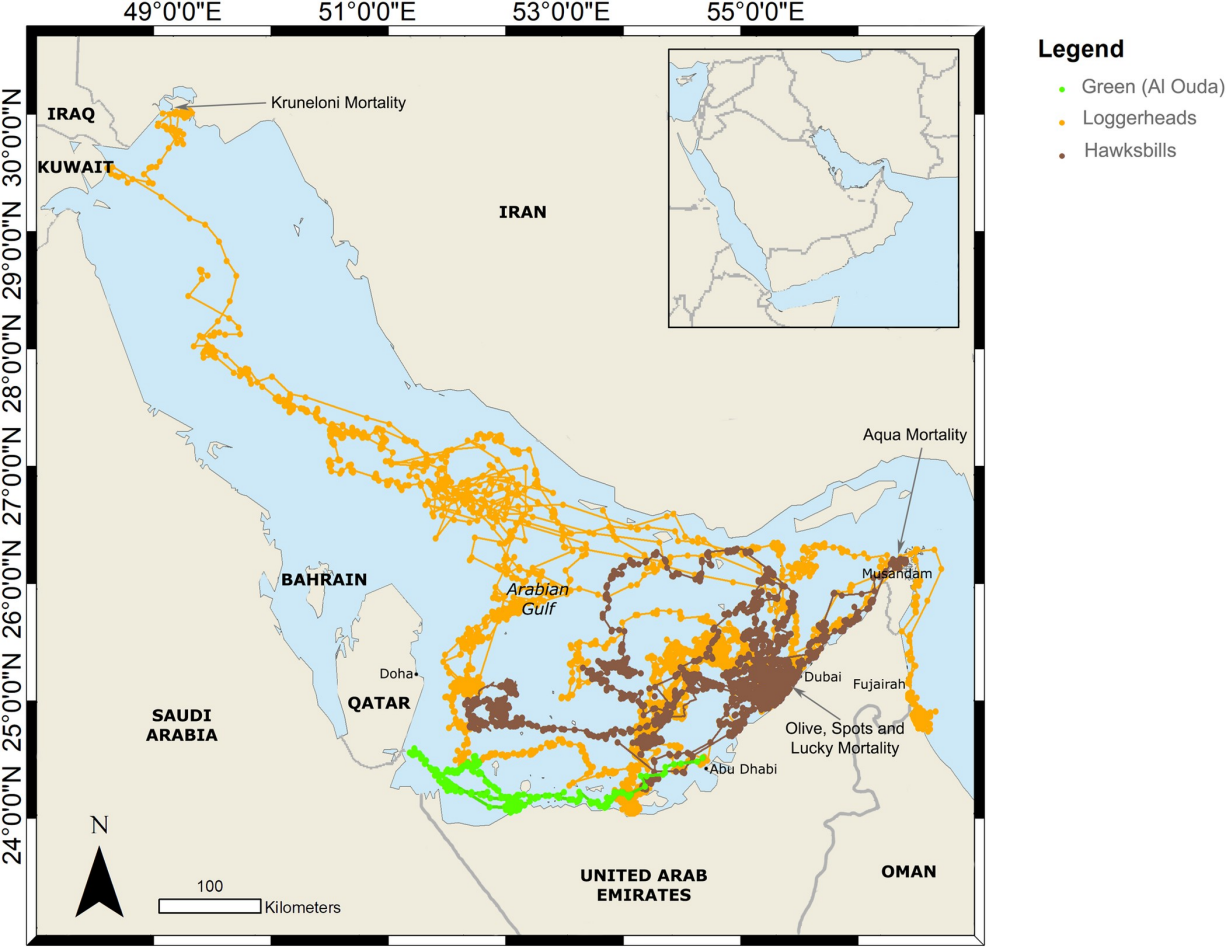
Horizontal movement by species of all rehabilitated sea turtles satellite tagged and released in the UAE, including locations where mortality occurred.

Amputee green turtle ‘Al Ouda’ was released from Abu Dhabi and travelled along the coastal waters of the western region of the UAE, entering Qatari waters towards the end of the tags transmissions. The green turtle ‘Al Ouda’ stayed in coastal waters of the western UAE and Qatar (Fig 3).

### Habitat use

**Fig 4:**
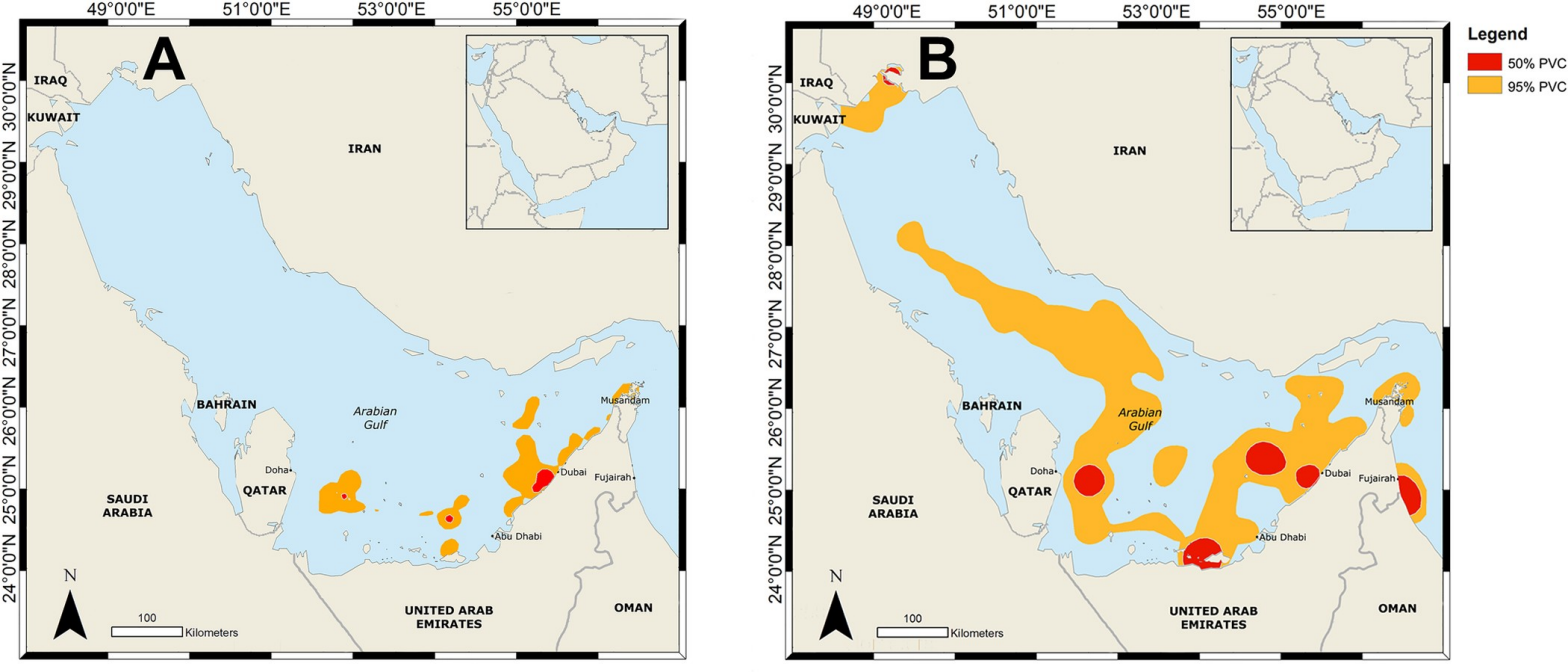
Combined 95% and 50% percentage volume contours for rehabilitated hawksbill sea turtles (A) and loggerhead sea turtles (B) that were satellite tagged and released in Dubai, UAE.

**Fig 5:**
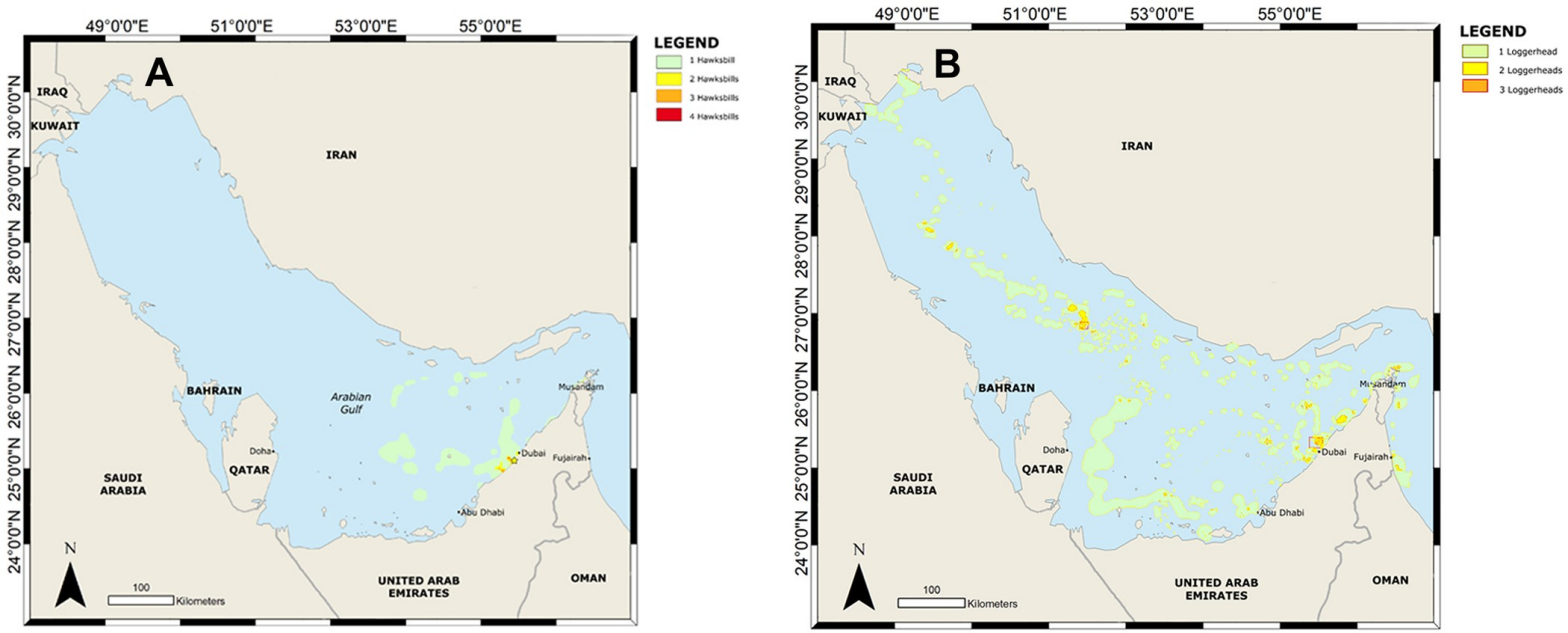
Overlapping home range usage for 11 rehabilitated hawksbill turtles (A) and 11 rehabilitated loggerhead sea turtles (B) released in Dubai, UAE.

Comparisons of the 95% PVC areas showed that hawksbill turtles had a relatively smaller home range extending not far from the release point (Fig 4A), whereas some loggerhead turtles dispersed widely, resulting in a larger home range (Fig 4B). The main core habitat (50% PVC) for hawksbill turtles was close to Dubai, with additional smaller core habitat areas in the shallow waters between Qatar and the UAE. Additionally, several of the tracked hawksbill turtles often used the same core habitat, underlining its importance. By contrast, the core habitat for loggerhead turtles was distributed over a larger area, with 50% PVC areas in coastal waters near Dubai as well as in deep waters of the Gulf and in the Gulf of Oman. These core habitats were largely made up by single turtles, with few small areas that had multiple turtles (Fig 5B).

#### Overlapping home ranges

Some areas were particularly important for released sea turtles. For example, the coastal area immediately around Dubai was used by up to four hawksbill turtles (Fig 5A). Loggerhead turtles only had small areas of overlap which were widespread from the Gulf of Oman, Strait of Hormuz, UAE coast and within the deeper areas of the central Gulf far from their release point (Fig 5B).

#### Mortalities

Over the combined 3,363 days of tracking 5 out of 26 sea turtles are thought to have suffered mortality. Three sea turtles (not included in the tracking analyses) were struck by boats soon after release and subsequently died. ‘Olive’, a sub-adult olive ridley turtle, was struck within four days of release and the tag didn’t provide adequate locations within that time to produce a map. Identification and cause of death were confirmed by visual observation of the carcass, with equally spaced deep lacerations indicative of a propeller. ‘Spots’, a sub-adult green turtle, and ‘Lucky’, a sub-adult hawksbill turtle, were both amputees and were released on the same day in November 2017. Both sea turtles swam into the man-made Dubai Canal (Fig 3), where they were killed by separate boat strikes which were also visually confirmed. Tagging results suggest that the loggerhead ‘Kruneloni’ died several months after release, after having formed its core habitat within the Shatt Al Arab (Fig 3), and that ‘Aqua’ (Fig 3) died after becoming entangled in a fishing net in the Musandam region of Oman. Aqua’s tag stopped transmitting as normal on 17 December 2017 then went into haul out mode four days later and, stopped transmitting soon after.

### Temperature

#### Hawksbill turtles

**Fig 6:**
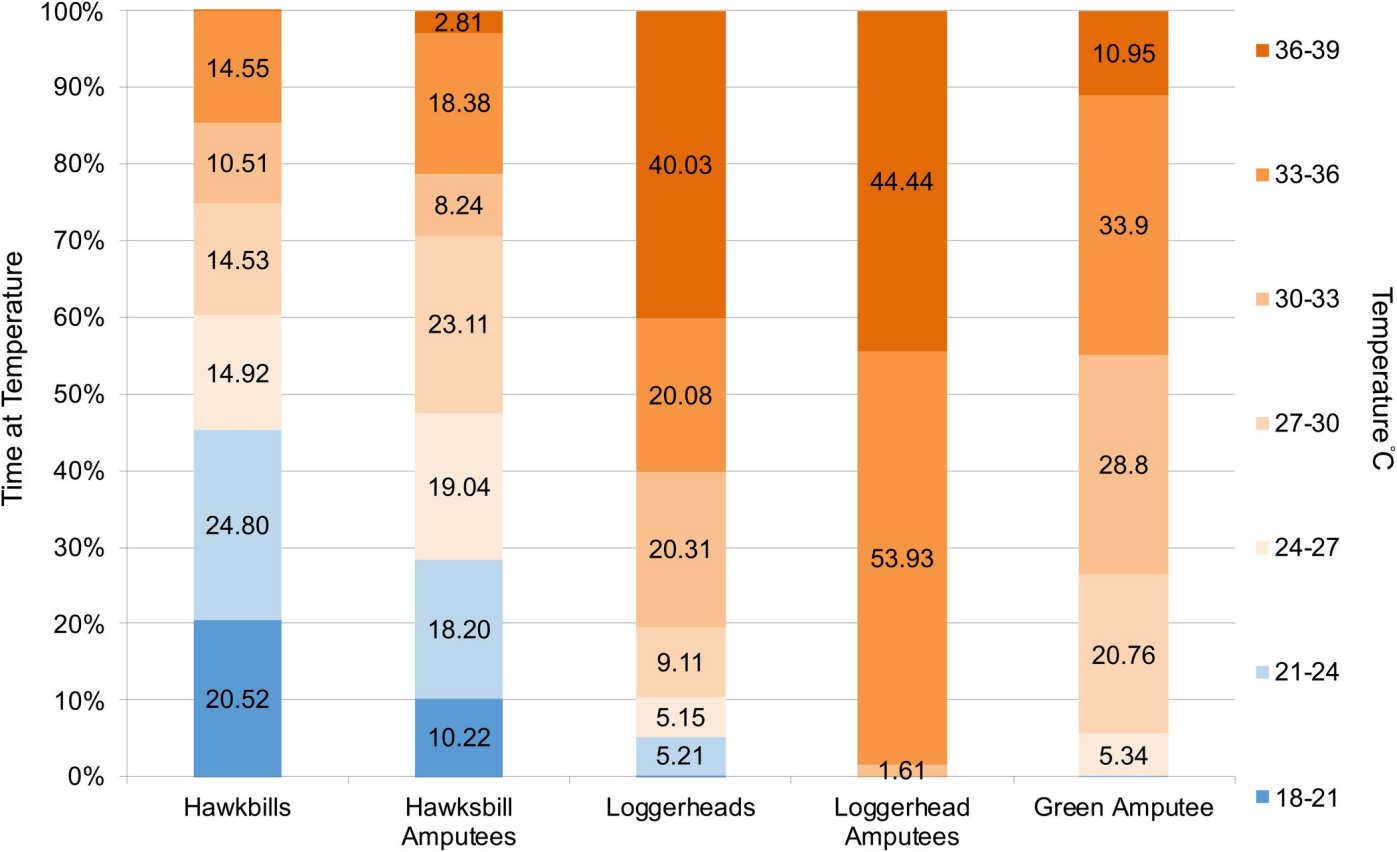
Composite annual percentage time-at-temperature histogram for hawksbill, loggerhead and green turtles, with and without amputations, for months when temperature was transmitted.

Water temperatures for all hawksbill turtles ranged from 18–36°C (Fig 6). Hawksbill turtles spent relatively even amounts of time in all temperature bins as data were transmitted throughout the year (Fig 6). The temperature distribution did not vary between amputees and non-amputees (*U*-value: 24; p-value < 0.05).

#### Loggerhead turtles

The temperature profile for the single amputee loggerhead turtle was similar to that of the non-amputees (*U*-value: 15; p-value < 0.05). We thus combined data from the amputee and non-amputee loggerhead turtles for all further analysis in this manuscript.

#### Green turtle

The temperature profile (24–39°C) for the single amputee green turtle was within the range of the non-amputee sea turtles from a previous study for months when temperature data were transmitted and comparable [5] and showed no significant difference (U-value: 19; p-value < 0.05).

## Discussion

### Threats to sea turtles in the Gulf

Our results confirm that rehabilitated sea turtles can be successfully released, for at least their tracking period, including those that have been in captivity for substantial periods due to serious injury or illness. We acknowledge that our home range estimates will have some artefacts because of differences in the number and quality of locations across individuals and in part due to the lack of funding (and suitability in the earlier years) for relatively expensive Fastloc-GPS tags as used in Thomson et al., [33] but, the key differences across individuals are likely still captured in this study and comparable for past and future studies. Twenty-one of the 26 released sea turtles survived in the wild following recovery from varying ailments, at least for the duration of their tracking. However, sea turtles face numerous anthropogenic threats, with boat strike and entanglement in fishing gear the two likely causes of death in the five sea turtles that were killed during our study. These causes of death show that the mortalities were likely to be unrelated to their ailment and rehabilitation, further underlining the success of this rehabilitation program. Hays et al. [30] analysed sea turtle satellite tracking data from around the world in an attempt to identify when sea turtles had been killed or captured and found a high level of suggested mortality. Cessation of tag transmissions were mostly caused by tag failure or battery depletion, but for some tags unusual behaviour suggested capture by fishers [30]. Wallace et al. [34] found that fisheries bycatch is the single greatest threat to sea turtles globally and noted that differing fishing rates around the world lead to differing mortality rates depending on the region of study. They also showed that propeller strikes are an important cause of death for sea turtles, corroborating our results from Dubai. Using a combination of photo-identification and satellite tracking technology in the Mediterranean, Schofield *et al*. [35] found an annual mortality rate of 0.11 for female and 0.27 for male loggerheads. Bentivegna *et al*. [36] found that in the Mediterranean the main causes of loggerhead decline was capture in drift nets and a reduction in nesting area. They also noted injuries caused by boat propellers, especially during the summer months when boat activity is high. While the impact of ingestion of fishing hooks and plastic bags in the Gulf has yet to be examined, one study highlighted that 87.5% of examined green turtles on the east coast of the UAE had ingested marine debris including plastic [37]. As with other regions, these threats are likely to lead to population declines in already low population sizes. Threats to sea turtles are similar in different regions of the world, but the importance of each threat category may vary. In UAE waters and the broader Arabian Gulf boat traffic and fishing cause most mortalities, as seen in our study. It is unlikely that the mortalities were linked to the sea turtles’ original injury and rehabilitation, but rather these cases demonstrate the threats faced by sea turtles in the Gulf region.

### Rehabilitation contribution

This study demonstrates that sea turtles continue to face many anthropogenic threats in Gulf waters even though most Gulf countries have laws in place to protect them. There are relatively small nesting populations of green and hawksbill turtles throughout the Gulf [9, 13, 25, 38] but, to the authors knowledge there are no reports of nesting activity by loggerhead turtles in the Gulf. Since its inception in 2004, the DTRP has treated and released over 1800 sea turtles that are likely to have died without human intervention (DTRP, unpublished data). Most of these sea turtles were juvenile hawksbills, a species that has low genetic variability in Gulf populations when compared with Indian Ocean populations, with an estimated 53 female hawksbills that contributed to hatchlings from three key nesting sites in the UAE [9]. We therefore believe that this rehabilitation program has been a positive and significant contribution to help support this regional sea turtle population.

The data collected from this study also contribute to the small body of literature from this region. Indeed, rehabilitation projects can give access to individuals of species, sex and life stages that are hard to access in wild populations [5] and therefore provide insight into the behaviour of other life-stages. For example, we tracked sub-adult and juvenile individuals, while most other tracking studies focus on mature, post-nesting females [39, 40]. This bias is because adult females are easy to access when they lay eggs on a beach. The same pattern exists for sea turtle studies in the Gulf region. Pilcher *et al*. [13] satellite tracked 90 post-nesting hawksbill females in the Gulf, but few tracking data are available for other species and other sexes and life stages. The only exceptions were from two studies on rehabilitated sea turtles. Rees *et al*. [41] tracked two rehabilitated and one post-nesting female green turtle. No clear difference was noted in the behaviour of the rehabilitated and non-rehabilitated sea turtles. Robinson *et al*. [5] satellite-tracked eight rehabilitated green turtles, including the longest tracked journey of a green turtle to date (8,283 km), and noted that behaviours appeared normal for all eight individuals. Our study adds new insight into sea turtle movement and habitat use in the Gulf area, particularly for juvenile and sub-adults and for loggerhead turtles that have not previously been tracked in this region.

#### Amputee turtles

Our tracking data showed that individuals with an amputation can be successfully rehabilitated. Both hawksbill and loggerhead amputee turtles behaved similarly to their non-amputee counterparts, with some even swimming further and dispersing faster than non-amputees. This is despite the fact that amputee turtles required much longer rehabilitation periods than non-amputees.

### Sea turtle habitat use

Several areas of important sea turtle habitat within the Gulf were identified through the tracking of four species and varying life-stages. The mean tracking duration was similar for loggerhead (161 days) and hawksbill turtles (131 days), but the habitat use varied greatly between the two species. The core habitat for hawksbill turtles was in the coastal area between Dubai and Abu Dhabi. Some areas within this core habitat were utilised by up to four individual hawksbill turtles. Many of the tracked adult female hawksbill turtles also moved to this area after nesting [14], further underlining its importance to the species. Tracked rehabilitated green turtles also heavily used this same area [5], which makes it an ideal location for targeted management applications to improve protection for turtles in the UAE. This area is made up of coral reefs and seagrass beds which are ideal feeding grounds for these two species [23].

Loggerhead turtles had a much larger home range than hawksbill turtles, with individuals moving as far as Iraq and into the Gulf of Oman. The main area of overlap among tracked individuals was in the central Gulf between Saudi Arabia and Iran. Their farther dispersal may be a function of their feeding ecology. Hawksbill and green turtles rely on seagrass and coral habitat to feed, while loggerhead turtles feed on a wider variety of prey, enabling them to move into other habitats as well. Furthermore, the relatively larger home range was likely because loggerhead turtles, unlike hawksbill turtles, are thought to transit through rather than breed in the Gulf. Nearby, however, large populations of nesting loggerhead turtles exist on the Indian Ocean coastline of Oman [42]. Only two of the eleven tracked loggerhead turtles moved out of the Gulf, but neither moved to the known nesting areas, instead forming core habitat close to the UAE/Oman border. The fact that most loggerhead turtles, including adult females, did not exit the Gulf may indicate that they forage widely for extended periods within the Gulf. Alternatively, it is also possible that they nest within the Gulf, and the area around Shatt Al Arab in particular may be of interest for future research.

## Conclusions

A clear need exists to assess the contribution of rescue and rehabilitation to sea turtle conservation. Here, we have shown that sea turtles from multiple species can successfully survive in the wild following extensive periods of rehabilitation. The relatively high level of mortality post-release is thought to be related to human threats within the Gulf, rather than to the sea turtles’ previous injuries or the rehabilitation process. Godley et al. [39], questioned the normality of post-rehabilitated sea turtles’ behaviour, and therefore their suitability for inclusion in satellite tracking and behavioural studies. This study does provide some support for rehabilitated sea turtles, even those who have undergone long periods of care and/or with serious injuries such as amputations, as potentially valid candidates for inclusion in behavioural analyses. This study also supports the suitability for release of sea turtles that have suffered major injuries, such as amputations, back into the wild. This is particularly relevant to life-stages such as male sea turtles and juveniles, which have rarely been included in movement analyses. Rehabilitated sea turtles displayed normal behaviours and returned to known and some potentially new foraging habitats. We could not test whether the rehabilitated adult sea turtles resumed breeding, due to a lack of long-term tag attachment. Investigating their return to nesting beaches would be a logical next step for sea turtle rehabilitation tracking studies.

As human populations grow, the number of sea turtles being injured or becoming sick due to anthropogenic reasons will likely also increase. Where possible, the implementation of rehabilitation projects can provide a valuable counterbalance for enhancing the welfare of individual sea turtles, and potentially help to mitigate population declines. The successful wild survival of the majority of sea turtles tracked here is an encouraging result for the conservation of this threatened group.

## Supporting information

S1 Fig (a) Horizontal movements of six of the rehabilitated hawksbill turtles satellite tagged and released in the UAE during this study. (b) Horizontal movements of the remaining five rehabilitated hawksbill turtles satellite tagged and released in the UAE during this study.
(TIF)

S2 Fig (a) Horizontal movements of six of the rehabilitated loggerhead turtles satellite tagged and released in the UAE during this study. (b) Horizontal movements of the remaining five rehabilitated loggerhead turtles satellite tagged and released in the UAE during this study.
(TIF)

S3 Fig (JPG)

S4 Fig (JPG)

S1 Data The individual post-filtered satellite transmitted data for each sea turtle included in this study. (XLSX)
