# Supplementary figures and images for: Satellite tracking of rehabilitated sea turtles suggests a high rate of short-term survival following release
Source: PLoS One. 2021 Feb 16;16(2):e0246241. doi: 10.1371/journal.pone.0246241 (PMC7886132; doi:10.1371/journal.pone.0246241)

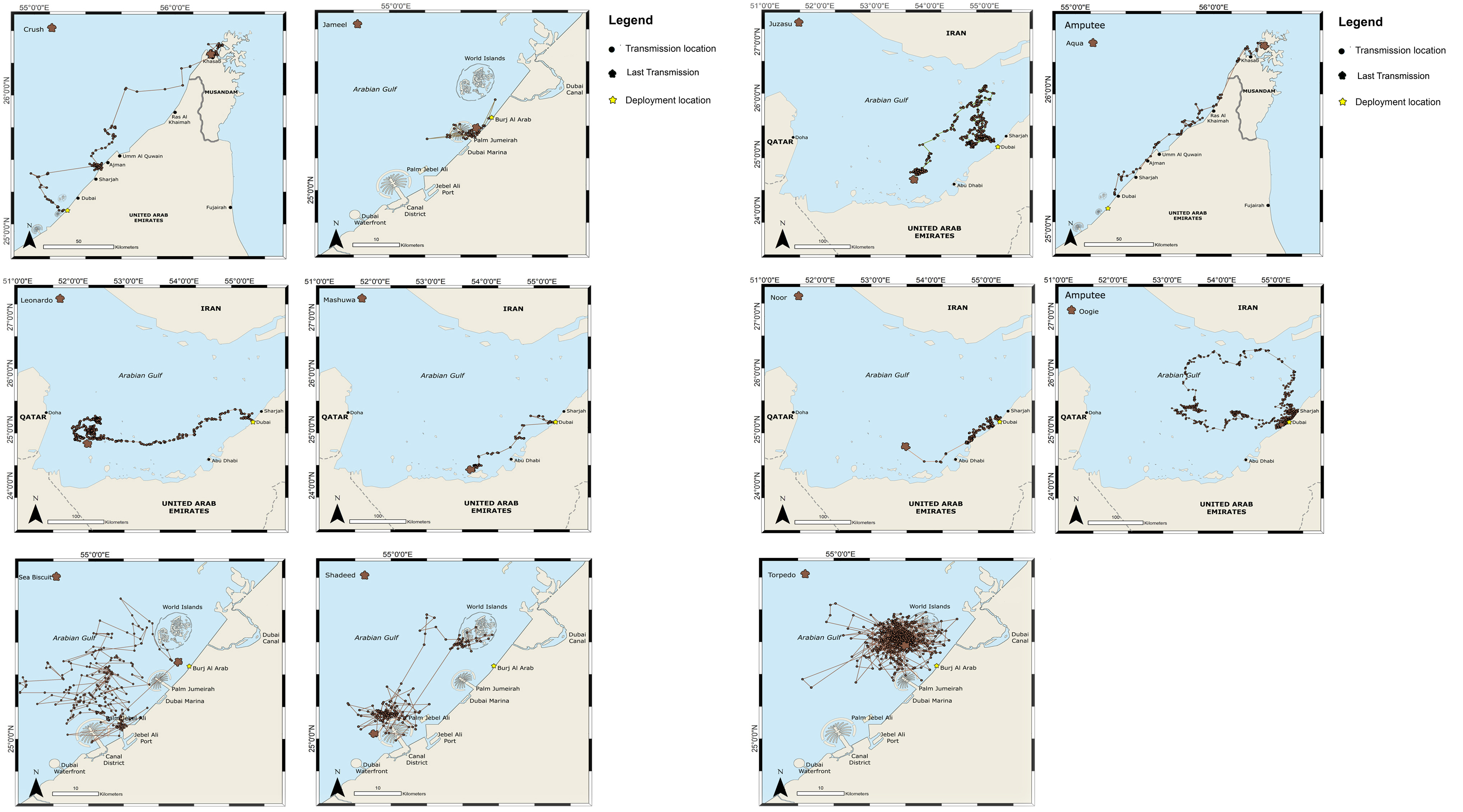

Supplement: S1 Fig — (a) Horizontal movements of six of the rehabilitated hawksbill turtles satellite tagged and released in the UAE during this study. (b) Horizontal movements of the remaining five rehabilitated hawksbill turtles satellite tagged and released in the UAE during this study. (TIF) [file pone.0246241.s001.tif]

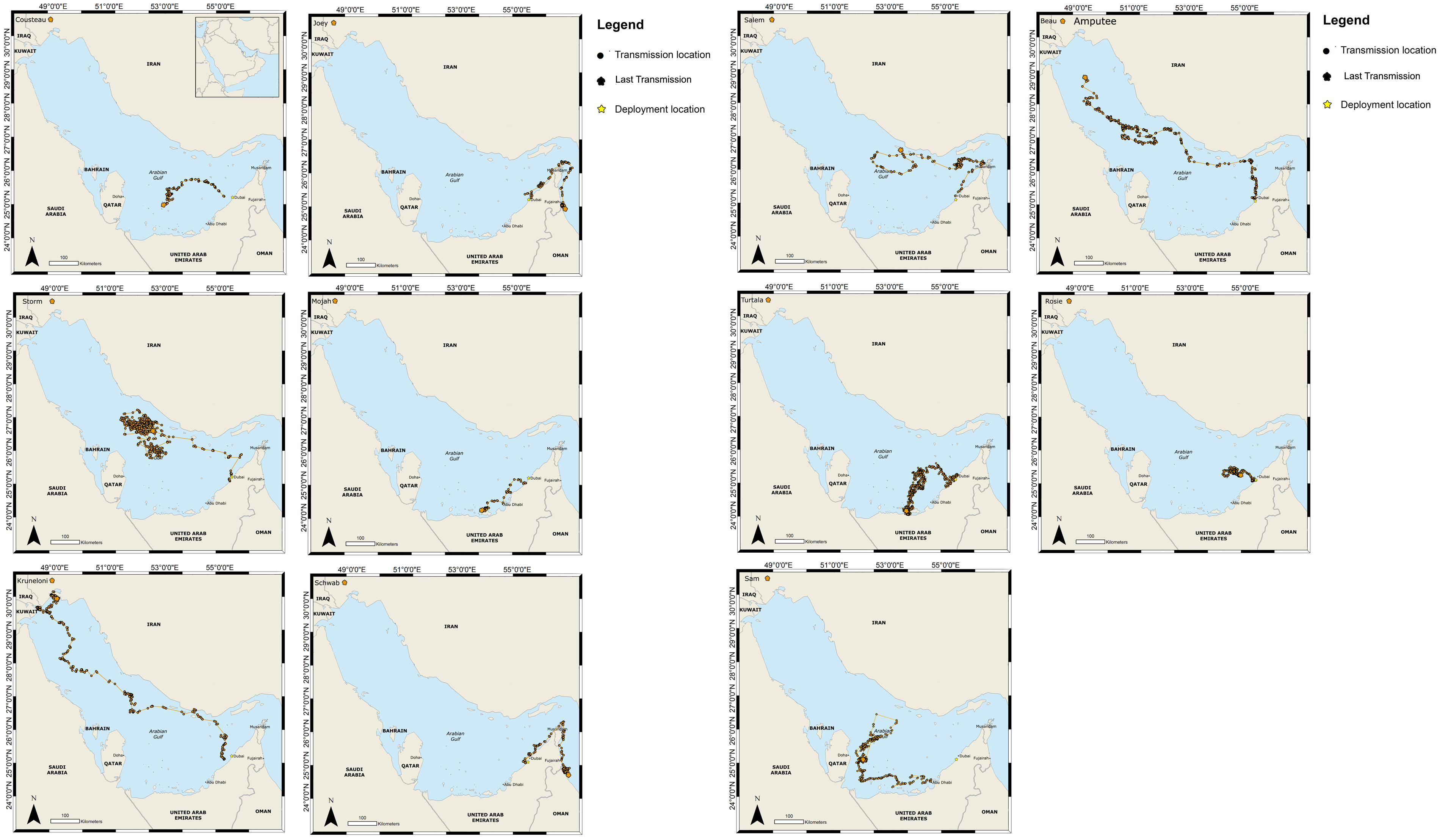

Supplement: S2 Fig — (a) Horizontal movements of six of the rehabilitated loggerhead turtles satellite tagged and released in the UAE during this study. (b) Horizontal movements of the remaining five rehabilitated loggerhead turtles satellite tagged and released in the UAE during this study. (TIF) [file pone.0246241.s002.tif]

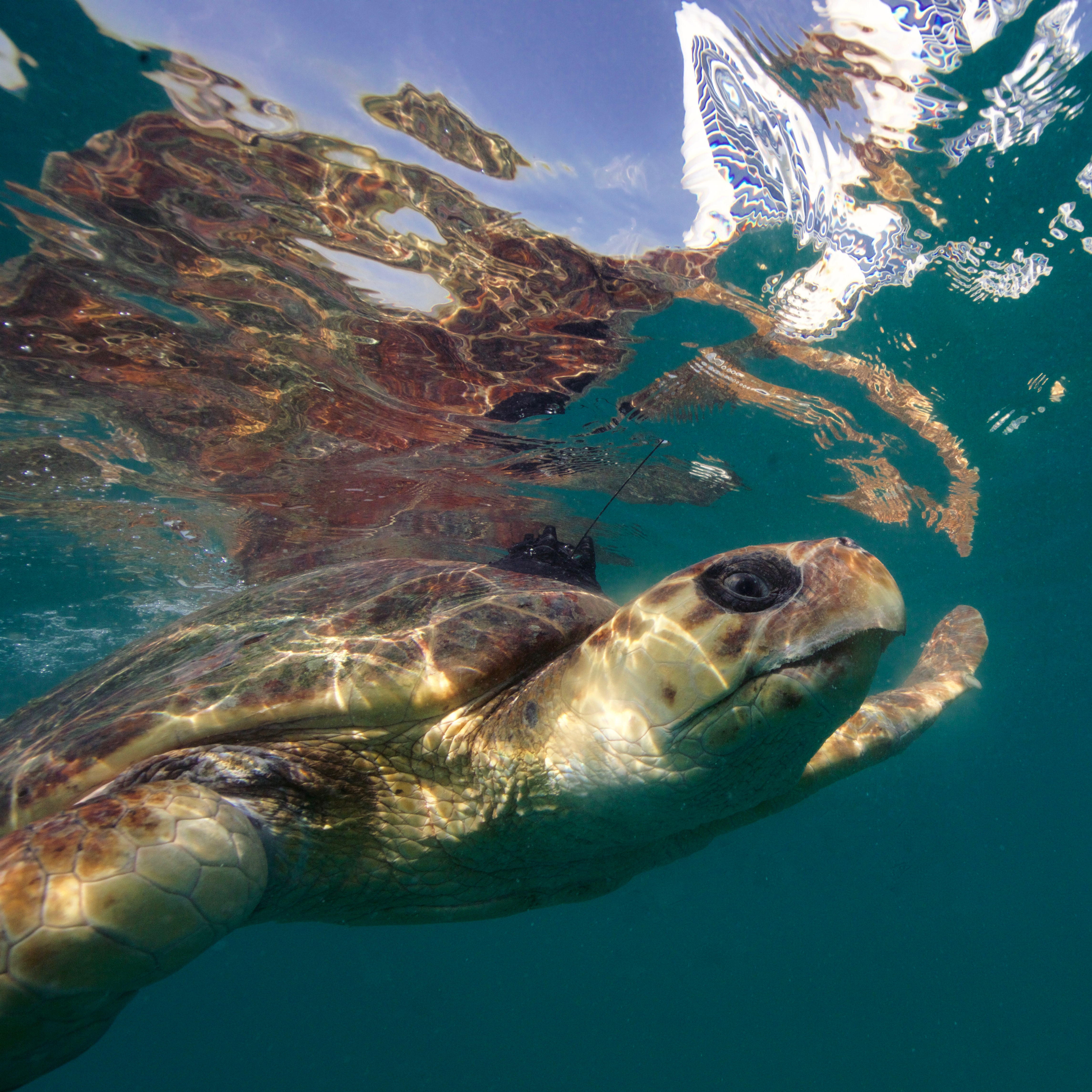

Supplement: S3 Fig — (JPG) [file pone.0246241.s003.jpg]

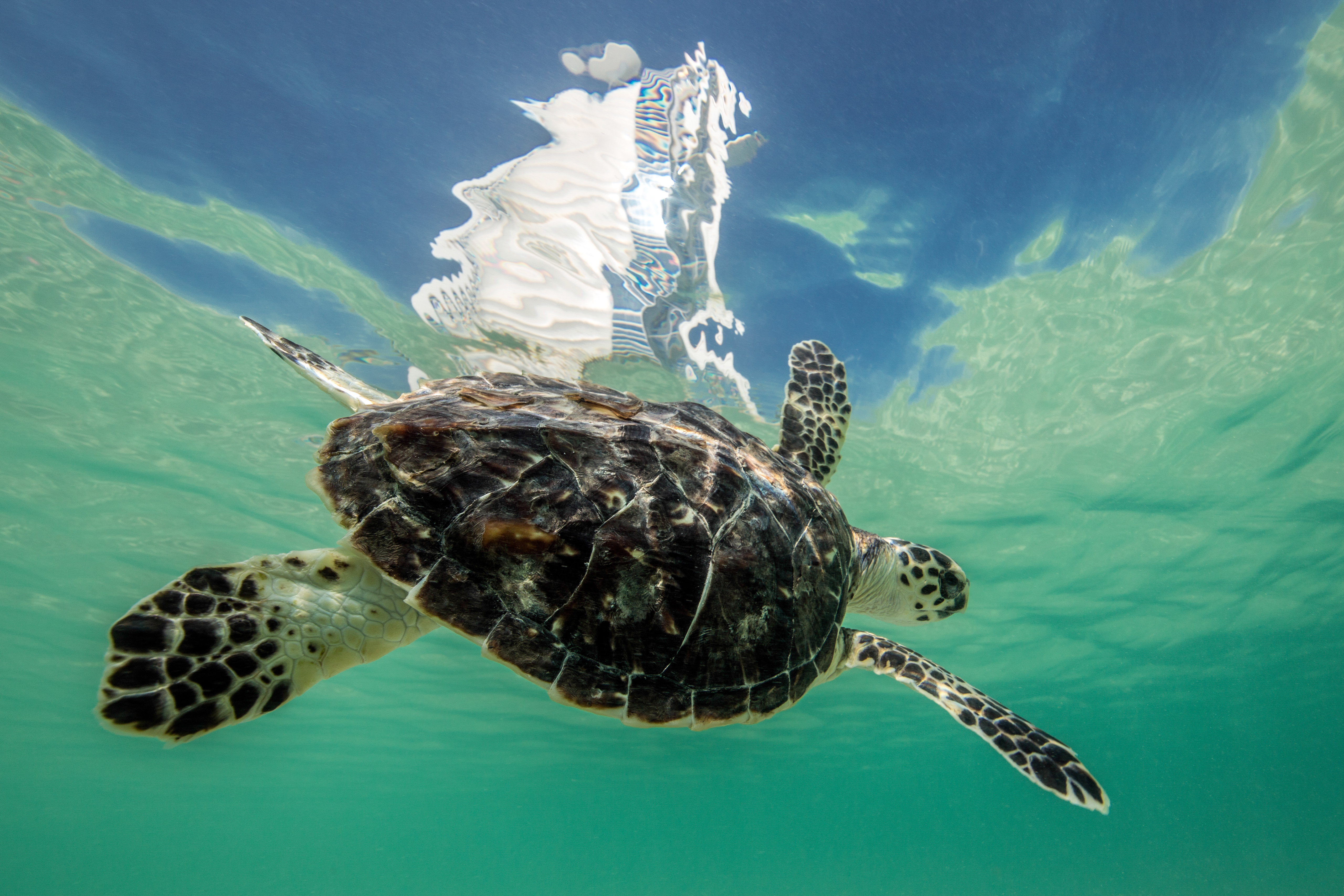

Supplement: S4 Fig — (JPG) [file pone.0246241.s004.jpg]
